# Na Battery Electrolytes Prepared by Dissolution of Commercial Polymers in NaPF_6_–Diglyme

**DOI:** 10.1021/acsapm.6c00803

**Published:** 2026-04-16

**Authors:** Ángela Campo, Nuria García, Ana López-Cudero, Aram Hall, Reza Younesi, Pilar Tiemblo

**Affiliations:** † Instituto de Ciencia Y Tecnología de Polímeros, 16379 ICTP-CSIC, Calle Juan de la Cierva 3, Madrid 28006, Spain; ‡ Department of Chemistry − Ångström Laboratory, 8097 Uppsala University, Uppsala 75121, Sweden

**Keywords:** Na battery, solid electrolyte, NaPF_6_, diglyme, polymer gel electrolytes, electrochemical stability, Prussian white cathode, hard carbon

## Abstract

The incorporation of small amounts of polymers into liquid electrolytes to produce gel electrolytes has many benefits. Decreasing, or even avoiding flow reduces accidents caused by leaks, allows for simpler and more flexible geometries and configurations, stabilizes electrochemical cycling, and may even permit the separate recycling of each cell component. Direct mixing of polymers with liquid electrolytes is very frequently possible, and it is a sustainable and scalable procedure that avoids evaporation stages and produces thermoreversible materials. In this work, some of the polymers most commonly used for gel electrolyte formation—polyvinylidene fluoride (PVDF), polyvinylidene fluoride-*co*-hexafluoropropylene (PVDF-HFP), polyvinyl chloride (PVC), poly­(methyl methacrylate) (PMMA), and poly­(ethylene oxide) (PEO)—are employed to produce gels with the electrolyte NaPF_6_ 1.1 m in diglyme, and their behavior and performance are described. All these polymers are soluble in the electrolyte, and the ultrahigh molecular weight (UHMW) PEO and the halogenated polymers are able to form self-standing gels at concentrations ranging from 5 to 10 wt %. The fluorinated polymer gels left standing in an Ar glovebox progressively become colored, turning almost black after one month, showing a lack of long-term chemical stability. PVC 10 wt % and UHMW PEO 5 wt % gels, however, become hard gels with no macroscopic phase separation and remain stable for periods of months. Their electrochemical stability against Na electrodes was tested, showing that the PVC gel is not stable, and only the UHMW PEO 5 wt % gels were further tested with Prussian white (PW) cathodes. The PEO gel electrolyte, without any physical separator, showed equivalent performance in PW||Na half-cells as the baseline electrolyte with a glass fiber separator, while also being self-standing and solid-like in consistency. The PEO gel electrolytes also showed applicability to PW||HC full cells. Moreover, after electrochemical testing, the coin cells with PEO gels were easily disassembled, and their components were recovered.

## Graphical abstract

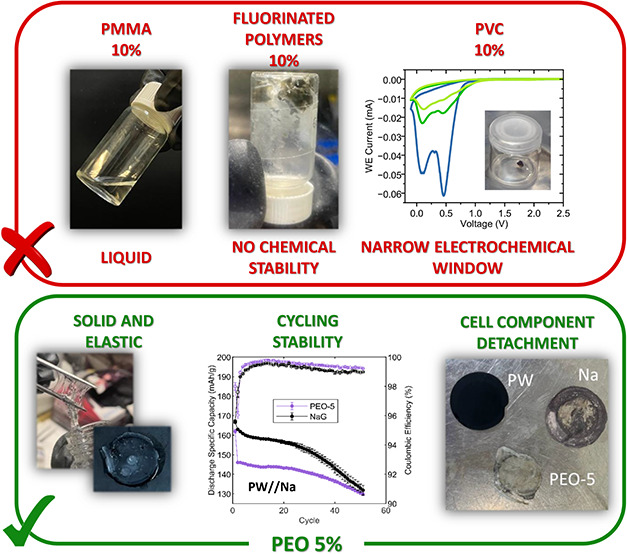

## 1 Introduction

The continuing electrification of transport has greatly driven up demand for battery energy storage. Though Li-ion batteries (LIBs) are technologically well-placed to meet this demand, many have reservations that enough extractable Li resources exist to meet this demand over the coming century. Sodium-ion batteries (SIBs) have been discussed as the drop-in replacement should Li costs rise, as they have a substantially similar structure and function to LIBs. Although they lag behind LIBs in areas such as energy and power density, they have the potential to outcompete LIBs in areas such as sustainability and cost. One area where SIBs hold potential benefits versus LIBs is in the choice of electrolytes.

Beyond the blueprinting of Li know-how, in the past decade, Na electrolytes have been paving their own way. Though approaches derived from Li are very often valid for Na, optimum choices for anions differ considerably, mainly because of the specific bonding characteristics of Na salts in comparison to Li, and their different sizes.^1^ Among Na salts (TFSI, FSI, BF_4_, ClO_4_, and PF_6_, all of them anions common to Li electrolytes), PF_6_ seems to be the most balanced candidate so far. Intercalation of solvated ions into graphite is accompanied by very notable irreversible reactions in the case of NaTFSI and NaFSI^2^ and both anions corrode the Al collector. At the Na electrode, NaTFSI and NaClO_4_ produce instable interfaces, leading to decomposition reactions.^2^ BF_4_ salts are slightly soluble, difficult to dry, and lack sufficient purity.

Among solvents, cyclic carbonates produce the best combination of stability and highest conductivities, but glymes have also proved to be an option.^3^ Glymes are excellent solvents for Na salts, and though their oxidation potential is low, the Na-complexed glymes show higher electrochemical stability^3^ due to the shift in the HOMO level. Furthermore, they display excellent stability against sodium metal,^4^ are able to intercalate into graphite electrodes,^5^ and perform well in cathodes,^6^ particularly diglyme. Therefore, the NaPF_6_ solution (∼1 m) in diglyme is a plausible electrolyte for commercial Na batteries.

In Na secondary batteries, polymer gels offer the same advantages as in Li, Al, or Zn. Flow control reduces the risk of leaks and allows for simpler and more flexible cell geometries and configurations, and gels have been seen to stabilize the electrochemical performance upon cycling. Solid-like electrolytes may even permit new ways of recycling by cell components. Literature on polymer gels of Na electrolytes is still not as large as on Li electrolytes, though attempts to prepare polymer gel electrolytes already exist.^7,8^ Typical preparation procedures with fluorinated polymers involve dissolution in DMF or in DMF/water mixtures, followed by casting or coagulating, as in the pioneering work in 2015 ^9^ and several others that followed. Polyacrylonitrile (PAN)^10^ and poly­(ethylene oxide) (PEO) have also been used because of their ability to form gels without chemical cross-linking. In the case of poly­(methyl methacrylate) (PMMA), other acrylates, methacrylates, or copolymers including methacrylic monomers, in situ polymerization and chemical cross-linking are usually employed.^11,12^

To enhance the mechanical properties of gels, several approaches include the use of a rigid scaffold such as cellulose, glass fiber, or silica particles coated by the polymer gel. For instance, cellulose and PEO electrolytes,^13^ chitosan and poly­(vinyl alcohol) (PVA),^14^ the coating of a porous cellulose membrane with cross-linked PMMA,^15^ the formation of a polyvinylidene fluoride-*co*-hexafluoropropylene (PVDF-HFP) coating by the non solvent-induced phase separation (NIPS) process around a glass fiber scaffold, multilayer cellulose-based separators coated by electrospinning of PAN with alumina particles,^16^ or gel polymer electrolyte nanocomposites based on PMMA and dispersed with silica nanoparticles^17^ are documented in the literature. Recent reviews compile the lines of research in this field, where the polymers employed are PAN, PVC, polyvinylpyrrolidone (PVP), fluorinated polymers, and PEO, and the fillers comprise MOFs, COFs, silica or other metal oxides, NASICON, or sulfides.^18^

The strategies listed above employ auxiliary solvents, with DMF being the most frequently used. Dissolution of electrolytes and polymers in organic solvents, followed by casting of the solution and solvent evaporation, has long been the main procedure used to prepare polymer gel electrolytes or polymer electrolytes. In polymer science, casting is avoided whenever possible since the complete elimination of solvent molecules is not possible, and it is a procedure burdened with intrinsic sustainability (and scalability) issues. In the literature, the alternatives to casting (or the NIPS procedure) are complex in situ polymerization and chemical cross-linking approaches. Chemical cross-linking makes the gel electrolyte thermostable (not thermoreversible) and thus less interesting from recycling or recovery viewpoints. On the other hand , in situ polymerization in the presence of the electrolyte is not a simple process, and subproducts from incomplete reactions remain in the gel electrolyte.

Direct blending or dissolution of polymers in many different electrolytes is not only possible but also a much better option than casting since it does not require the use of auxiliary solvents, reduces the preparation time, and is more reproducible. Depending on the polymer/liquid electrolyte ratio, direct blending can be done by employing extruders or mixers, or by simple dissolution in the liquid electrolyte.^19,20^ Other additives, such as fillers, can be incorporated, producing composite polymer electrolytes.^21,22^

Obviously, polymer dissolution in the liquid electrolyte entails the existence of favorable interactions that can imbalance the species equilibrium of the neat electrolyte. This is very clearly seen when using deep eutectic electrolytes or deep eutectic analog electrolytes, where the dissolution of the polymer can even produce the precipitation of the deep eutectic blend components.^23,24^ As a matter of fact, in polymer gel electrolytes in general, and certainly in those prepared by direct dissolution, the polymer’s role is that of an active species in the electrolyte, and the changes in speciation of the gel electrolyte should always be considered.^20,23^

In this work, we explore the formation of polymer gels with 1.1 molality (m) of NaPF_6_ in diglyme, employing a set of different polymers directly soluble in this electrolyte: PMMA, PVDF, PVDF-HFP, polyvinyl chloride (PVC), and PEO. To obtain an optimum balance between rheology and electrochemistry, the lowest content of polymer and the highest molecular weight (MW) are pursued. We study their ability to form self-standing gels and their interaction with the liquid electrolyte, paying special attention to chemical speciation or other structural modifications because of the polymer’s presence. Phase transitions are studied by XRD and DSC. The physical and chemical stability of the self-standing polymer gels over time, frequently overlooked, is also studied carefully. Ion diffusivity is characterized by PFG-NMR at 25 and 30 °C, and ion conductivity is studied across a large T range. The electrochemical stability of the self-standing gels obtained is studied in Prussian white (PW) PW||Na and PW||HC cells.

## 2 Experimental Section

### 2.1 Materials

Sodium hexafluorophosphate salt (NaPF_6_), high purity, was purchased from Fluorochem (Ireland), and diethylene glycol dimethyl ether, 99.8% anhydrous (diglyme), was purchased from Sigma-Aldrich (MO, USA).

PEO of M_V_= 80 × 10^5^ g mol^–1^, PMMA of M_W_= 1.2 × 10^5^ g mol^–1^, PVP of M_W_= 13 × 10^5^ g mol^–1^ and PVDF of M_W_= 5.3 × 10^5^ g mol^–1^ were purchased from Sigma-Aldrich (MO, USA). PVDF-HFP of M_W_= 3 × 10^5^ g mol^–1^ was purchased from Solvay (France), and PVC of M_W_= 0.6 × 10^5^ g mol^–1^ was purchased from Westlake (Spain).

For the electrochemical measurements, sodium metal electrodes were prepared by pressing Na onto Al foil inside an Ar-filled glovebox ([O_2_] < 1 ppm, [H_2_O] < 1 ppm). Pressed Na electrodes were punched at a 15 mm diameter. Prussian white (PW) electrodes were prepared using PW from Altris AB (Sweden). Electrode slurries were processed in deoxygenated water with a weight ratio of 85:10:5 for PW, C-ENERGY C65 (Imerys, France), and sodium carboxymethyl cellulose (CMC; 2200, Daicel, Japan), and were blended in a shaker with Al_2_O_3_ balls for 60 min at 30 Hz prior to coating. Electrode films were cast on carbon-coated Al foil at 200 μm thickness, yielding a mass loading of 2.3 ± 0.2 mg cm^–2^. Hard carbon (HC) electrodes were prepared similarly but cast at 100 μm thickness, yielding a mass loading of 1.0 ± 0.2 mg cm^–2^. After coating, the electrodes were dried on a heated plate at 55 °C, punched to a 13 mm diameter, and then immediately moved to the glovebox. The electrodes were then thoroughly dried under vacuum at 170 °C for 15 h. For the full cells, assuming a nominal capacity of 170 mAh g^–1^ for the PW and 300 mAh g^–1^ for the HC, the n:p ratio was 1.0 ± 0.2. Electrochemical stability window testing was performed in beaker cells with solid glassy carbon working electrodes (Goodfellow; 1 mm thick) using Na reference and counter electrodes.

### 2.2 Polymer Gel Electrolyte Preparation

All electrolyte preparations were carried out under an Ar atmosphere in a glovebox ([O_2_] < 1 ppm, [H_2_O] < 1 ppm). The liquid electrolyte was prepared by dissolving NaPF_6_ salt in diglyme. For better comparison with literature data, in which 1 m is usual, the initial molal concentration of the electrolyte was fixed at 1.1 m to account for the Na concentration decrease upon adding polymers. This electrolyte is called NaG hereafter. The solution was stirred vigorously at room temperature for 5 h. After complete dissolution, the solution was filtered using a 3 μm glass fiber (GF) syringe filter to ensure purity and then allowed to stand overnight before use.

Gel polymer electrolytes were prepared under an argon atmosphere in the glovebox. Initially, the desired polymer amount and NaG were mixed manually at room temperature for 1 min to achieve a homogeneous dispersion. The mixture was then gradually heated to 70 °C with continuous manual stirring for 10 min. For PVC, PVDF-HFP, and PVDF gels, this was followed by magnetic stirring at room temperature for 5 h to ensure uniform polymer dispersion. In contrast, PMMA dissolution did not require heating and was instead mixed manually at room temperature until a homogeneous solution was achieved. All polymer solutions and gels are listed in Table 1. The different polymer solutions are classified as liquids or gels depending on the time taken to flow when inverting a vial. Immediate flow characterizes a liquid.

**Table 1:** Nomenclature, Composition, Rheology, and Ion Mobility at 25 °C of the Electrolytes

			Ion mobility (25 °C)			
			D × 10^10^/(m^2^ s^–1^)			
Acronym	Polymer wt %	Rheology (inverted vial test)	^1^H	^19^F	^23^Na^a^	σ/(mS cm^–1^)
NaG	-		10.7	3.1	3.3	7.04 ± 0.61
PMMA-10	10	Liquid	6.7	1.8		4.48 ± 0.07
PVC-2	2	Liquid	9.3	2.6		6.09 ± 0.85
PVC-5	5	Liquid	8.6	2.2		5.74
PVC-7.5	7.5	Gel	8.2	2.1		5.34 ± 0.45
PVC-10	10	Gel	8.3	1.7		4.75 ± 0.20
PEO-085	0.85	Liquid	10.1	3.5		7.01
PEO-1	1	Liquid	9.7	2.8	3.1	6.84 ± 0.09
PEO-1.5	1.5	Liquid			3.2	6.96 ± 0.09
PEO-2	2	Liquid	9.7	3.2	2.8	-
PEO-5 (fresh)	5	Gel (elastic)	9.6	2.5		5.81 ± 0.54
PEO-5 (12h)	5	Gel (hard)				3.82 ± 1.66
PVDF-5	5	Liquid				6.02 ± 0.37
PVDF-10	10	Gel				5.26 ± 0.06
PVDF-15	15	Gel				4.03
PVDF-HFP-5	5	Liquid				6.47
PVDF-HFP-10	10	Gel				5.54 ± 0.06
PVDF-HFP-15	15	Gel				4.00

a D (^23^Na) at 30 °C.

### 2.3 Characterization

FTIR spectra were recorded in ATR mode using an FTIR PerkinElmer Spectrum-One (PerkinElmer, Waltham, MA, USA), with 4 scans and a resolution of 4 cm^–1^.

The differential scanning calorimetry (DSC) measurements were done in a PerkinElmer DSC7 instrument from TA Instruments. The heat flow was recorded from −80 to 80 °C at 10 °C min^–1^. First, a heating scan was done on samples prepared at least 24 h before, followed by a cooling scan and a second heating scan.

X-ray diffraction (XRD) measurements were recorded by using a Bruker D8 Advance diffractometer (Bruker AXS GmbH, Baden-Württemberg, Germany) equipped with a Vantec PSD detector. CuKα radiation (λ = 1.5406 Å) was generated at 40 kV and 40 mA. Data were collected with an angular step size of 0.025° (2θ) and a total acquisition time of 600 s at room temperature.

The diffusion coefficients for ^23^Na, ^19^F, and ^1^H were determined by Pulsed Gradient Solid State (PGSE) solid NMR. For the PFG NMR measurements, portions of the gels were placed in a 5 mm o.d. NMR tube (^19^F and ^1^H) or a 10 mm (^23^Na). The ^19^F and ^1^H diffusion data were acquired at 25 ± 0.1 °C, and those for ^23^Na were acquired at 30 ± 0.1 °C with a Bruker diffusion probe head, Diff50, using a simulated spin echo pulse sequence. The typical 90° radiofrequency (rf) pulse length for ^1^H and ^19^F was 1 ms, and the diffusion time was fixed at 20 ms. For ^23^Na pulse length was 0.7 ms, and the diffusion time was fixed at 10 ms. The scattering of diffusion coefficients is lower than ±15%.

The ionic conductivity (σ) of the electrolytes was measured by Electrochemical Impedance Spectroscopy (EIS) in a NOVOCONTROL GmbH Concept equipment with a high-performance frequency analyzer Alpha-A, in combination with a QUATRO Cryosystem, from −25 to 60 °C in the frequency range of 1–10^7^ Hz. All electrolytes were measured in the cell, as shown in Figure S1. Electrolytes were placed between two stainless steel electrodes with a diameter of 10 mm, covered by a Teflon scaffold with a precisely defined thickness of 700 μm to avoid creep during measurement. The lid of the cell, which incorporates this spacer, is simply placed over the lower electrode so that the spacer sets the exact electrode separation. This lid has two small holes to allow the evacuation of excess product. This design ensures a constant and reproducible thickness for all samples and is fully compatible with solid, gel, and liquid electrolytes. The measurement protocol includes three temperature ramps with data taken every 10 °C: first heating from 20 to 60 °C, then cooling down to −25 °C, and a final heating cycle up to 30 °C.

Electrochemical stability window of the electrolytes was measured using an Autolab PGSTAT302 potentiostat/galvanostat controller (Metrohm AG, Herisau, Switzerland), as shown with cyclic voltammetry. The gels were cycled in a range from 0 V vs Na^+^/Na to 2.5 V vs Na^+^/Na and from 2.5 V vs Na^+^/Na to 5 V vs Na^+^/Na using glassy carbon as the working electrode at a scan rate of 20 mV s^–1^.

Electrochemical performance tests were carried out by using a Neware cycler. Coin cells (2032 nm format) were assembled for all measurements. In the case of the gel electrolytes, the material was sandwiched between the electrodes using a polypropylene (PP) ring of 16 mm diameter, with a 1.1 mm thickness, and an 11 mm diameter hole as a mechanical spacer, without an additional physical separator. For liquid electrolyte cells, a Celgard separator was employed. The mass of active material was based on an average of 16 electrodes (3.0 ± 0.2 mg of PW per electrode). For the PEO-based gels, the entire active mass was considered, not just the area exposed by the PP ring. All cells underwent a formation cycle at a constant current rate of C/10 based on the theoretical capacity of 171 mAh g^–1^ for PW. All cells were then cycled at C/3 for 50 cycles. In PW//Na cells, the voltage window was 2 to 4 V, and for PW//HC cells, the voltage window was 1.8 to 3.8 V.

## 3 Results and Discussion

### 3.1 Polymer Gels Preparation and Physicochemical Characterization

PVP, PVC, PVDF, PVDF-HFP, PEO, and PMMA have been tested because they are among the polymers most commonly employed for solid Na electrolyte preparation. All except PVP (which is insoluble in NaG) are soluble in NaG at least up to 10 wt %. The rheology of the polymer solutions has been qualitatively studied at room temperature using the inverted vial test, and the results are presented in Table 1. Figure 1a shows images of the self-standing gels prepared with a minimum polymer content: PEO-5, PVC-10, PVDF-10, and PVDF-HFP-10. None of the non-UHMW polymers produce gels at concentrations under 7 wt %. PVC, PVDF, and PVDF-HFP produce gels starting from 7 wt % and self-standing ones at 10 wt %. In contrast, PMMA does not produce gels even at concentrations as high as 10 wt %.

**Figure 1:**
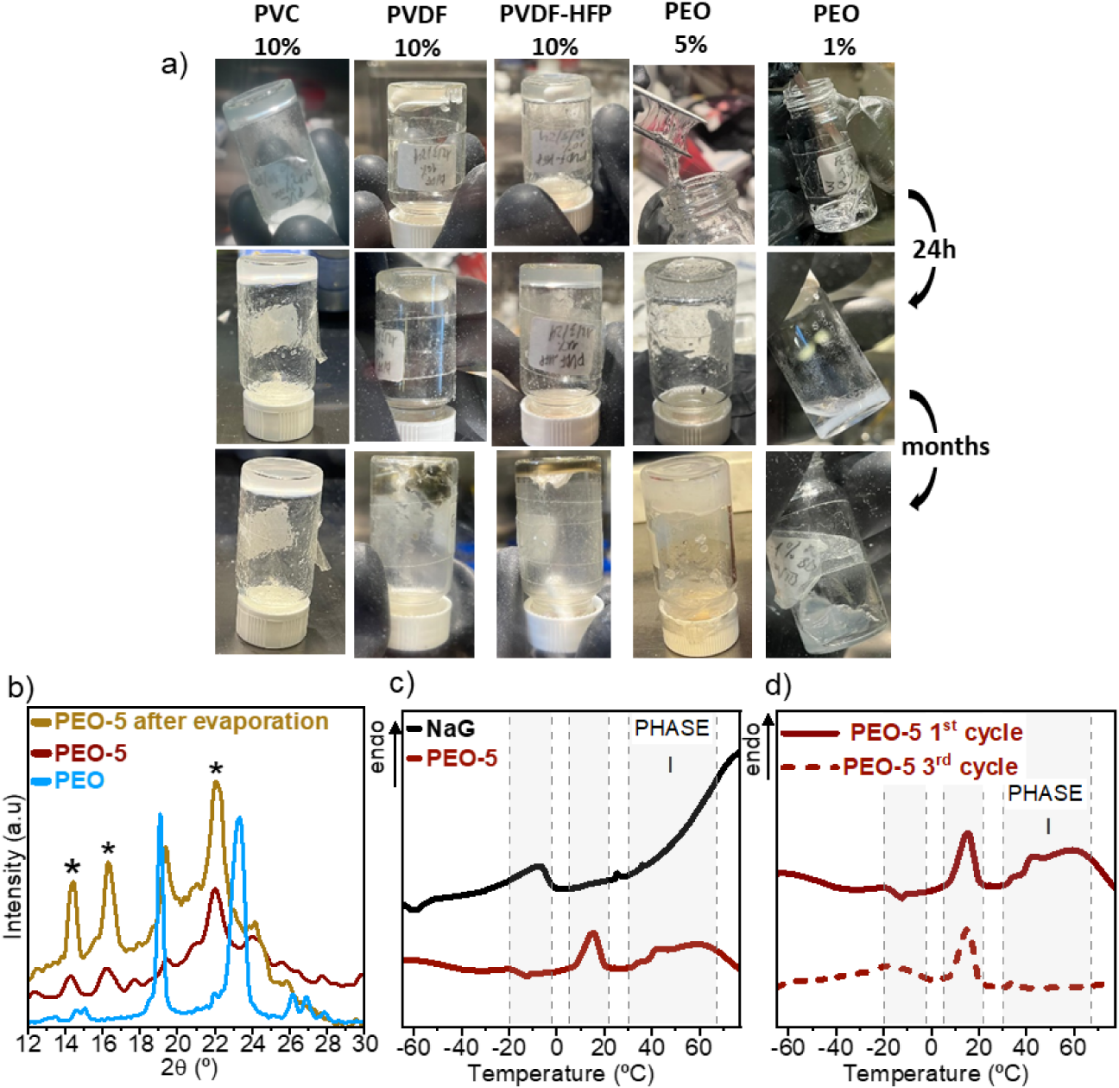
(a) Dimensional and chemical stability over time of the self-standing gels and PEO-1, (b) XRD of neat PEO, PEO-5, and PEO-5 after evaporation, showing the peaks at 14°, 16°, and 22° ascribed to PEO/Na crystalline complexes, (c) melting peaks in the first DSC cycle of NaG and PEO-5, from −60 to 70 °C, and (d) first and third cycles of PEO-5 gel from −60 to 70 °C.

UHMW PEO produces a self-standing gel, according to the inverted vial test, at 5 wt % (Figure 1a). This is a consequence of the well-known increase in the number of entanglements between chains as molecular weight increases. PVDF and PVDF-HFP are able to produce thermoreversible gels in many electrolytes because of their semicrystalline characteristics.^25^ In turn, PVC is well-known for its ability to form thermoreversible gels with many solvents. In this case, the physical cross-linking sites are specific configurations in the polymer, which produce interchain interactions.^26^ It cannot be excluded that the interaction of PVC with Na cations also contributes to the formation of the gel. Some authors have suggested an interaction of PVC with the Na salt NaBF_4_, where the ν­(C–Cl) of the gauche bond (600–640 cm^–1^) is perturbed and decreases in intensity.^27^

Specific interactions between NaG and the polymers have been studied by FTIR, and they are detailed in the Supporting Information file, where Figure S2 collects the FTIR spectra of NaG and its polymer gels. Figure S2a shows the FTIR spectra of PEO, diglyme, and NaG. PEO and diglyme differ only in the presence of bands associated with the methyl group in the diglyme and of bands related to long oxyethylenic sequences in PEO, such as 1060 cm^–1^, associated with TGT helical conformations and present in the crystalline phase. Also, the wagging ω­(CH_2_) is in diglyme at 852 cm^–1^ and in PEO at 841 cm^–1^, downshifted because of crystallinity and ordered sequences. The modifications in the diglyme spectrum as a consequence of the solution of NaPF_6_ are well seen: a strong intensity increase of the band at 852 cm^–1^ and a strong downshift to 838.5 cm^–1^, appearance of a narrow and intense band at 550 cm^–1^ and the splitting of the band at 1100 cm^–1^ into two bands. These modifications in the spectra have been studied before^2^ and are a consequence of the complexing of Na^+^ by the oxyethelenic units of the diglyme. This complexation is very strong and of the same nature as the one that occurs when dissolving metal salts (Na, K, Li, etc.) in PEO, since chemically, PEO and glymes are identical except for the chain ends. Figure S2b collects the spectra of NaG, PVC-10, PEO-5, PMMA-10, PVDF-10, and PVDF-HFP-10 in a broad IR region, showing the bands characteristic of the different polymers. Particularly interesting is the carbonyl band of PMMA, which is at 1732 cm^–1^, with no shifting, because of interactions with the Na cation. The lack of strong specific interactions between the polymer and the electrolyte, the amorphous character of PMMA, and its conventional molecular weight (not UHMW) explain the failure of this polymer to produce physical gels at concentrations under 10 wt %. This is why methacrylate gels reported in the literature are never physical but chemical.^11,12^ As mentioned in the introduction, chemical cross-linking produces thermo-irreversible materials, which cannot be reprocessed, and this is to be avoided as far as possible. Since there is no commercial UHMW PMMA, this polymer has not been considered further in this work for its use in the electrochemical cell.

In the PVC gel spectra shown in Figure S2c, the region of the ν­(C–Cl) increases in intensity as the PVC concentration in the gel increases. Figure S2d shows the upshifting, slight but clear, of the ω­(CH_2_) band of the PVC gels with respect to NaG. This shifting is opposite to the additive contribution of pure PVC ω­(CH_2_) bands and suggests the participation of Cl–Na interactions in the gel formation. In turn, the fluorinated polymer gels display bands corresponding to the CF_2_ group, with increasing intensity as the polymer concentration increases, whether PVDF or PVDF-HFP, as shown in Figure S2e.

After 24 h, the halogenated polymer gels showed no alterations, as shown in Figure 1a. However, after 24 h, the PEO-5 gel had hardened considerably, turned translucent, and a slight segregation of the liquid electrolyte was seen. At lower polymer content (0.85, 1, 1.5, 2 wt %), all freshly prepared PEO gels were sticky, soft, and elastic, which phase-separated into a white precipitate and a transparent liquid after 24 h, as shown in Figure 1a. These changes are thought to be caused by the crystallization of Na/glyme/PEO complexes, which precipitate unless the PEO content is high enough to form a percolated crystalline network, such as that in PEO-5 (Figure 1a). Crystalline complexes of PEO and metal salts have been known for long.^28^ The crystallinity of the gels in this work was studied by XRD and DSC. Figure 1b shows the XRD of neat PEO, PEO-5, and PEO-5 after evaporation, in order to more clearly see the peaks corresponding to the crystalline complexes. The peaks at 14° and 16° belong to the crystalline complexes of oxyethylenic units with Na, as reported by other authors^29^ whereas the peaks at 18° and 23°, overlapping with the amorphous halo, belong to PEO.

Figure 1c shows the melting peaks of NaG and its PEO-5 gels on a very expanded scale, because the existing crystallinity is extremely low in all of them. For the sake of comparison, Figure S3 shows a DSC from −80 to 80 °C of PEO, NaG, PEO-1, and PEO-5. Crystallinity is seen in either the liquid electrolyte or its gels with PEO, but it is very small (∼2–5 wt %) compared to that of pure PEO (about 60 wt %). This very low value is remarkable since, at room temperature, the macroscopic appearance of the gels with 5 wt % PEO content is that of a self-standing solid (as shown in Figure 1a) although more than 95 wt % of the material is in the molten state, i.e., a liquid.

The NaG liquid electrolyte has a melting peak at −7 °C (Figure 1c). The addition of only 1 wt % UHMW PEO to the liquid electrolyte causes this peak to decrease and disappear in gels with 5 wt % PEO, while new crystalline phases are seen, melting between 0 and 20 °C and over 40 °C. According to the literature,^30^ there are two crystalline phases in Na/PEO complexes: one that melts at about 40 °C (Phase I) and another melting from 80 to 90 °C (Phase II). Thus, the endotherm seen over 40 °C in the gel electrolytes is presumably related to Phase I Na/PEO complexes, while the peak at about 20 °C may belong to complexes of Na with both diglyme and PEO. Figure 1d shows the first and second DSC scans of PEO-5. In comparison to the first heating scan, during the second scan, the endotherm attributed to Phase I PEO/Na is not seen, and only the low melting T phases appear. This is very likely due to the fast cooling, which favors crystallization in low-viscosity environments (glyme-rich) rather than in high-viscosity ones (polymer-rich).

The formation of PEO/Na crystals in Na salt gel electrolytes does not always occur. For instance, we have checked that in PEO gels of Na bis­(trifluorosulfonyl)­imide (NaTFSI) 1 m in diglyme and 1 wt % PEO, no crystallization is seen. As shown in Figure S4, after several weeks, the gel electrolyte remains transparent and retains its characteristic elastic gel morphology. In PEO/M^+^ gel electrolytes, the crystallization of PEO/M^+^ complexes depends very strongly on the salt anion. Anions that are soft bases have a weaker interaction with the cation and plasticize PEO, making its crystallization more difficult. Hence, PEO gels of TFSI or FSI salts display higher ionic conductivity than those of harder bases. The basicity of the fluorine anions increases in the following order: TFSI > FSI > OTf > BF_4_ > PF_6_, and in PF_6_ electrolytes, PEO is expected to crystallize more than in TFSI ones. This PEO crystallization, which in polymer electrolytes may be a drawback, can be an advantage in gels with a large ratio of liquid electrolytes, as in this case. With as low as 5 wt %, PEO-5 is self-standing and remarkably hard. The crystalline phase in the gel can play the role of cellulose or glass fibers, or silica particles employed in the literature to confer mechanical strength to the gel electrolytes. The PEO/Na^+^ complexes produced in the polymer electrolytes presented in this work can be seen as a bottom-up rigid scaffold that allows to prepare electrolytes with enough ion conductivity and sufficient mechanical stability.^13,15,17^

### 3.2 Ion Mobility: Conductivity and Diffusivity

The ion conductivity of the polymer solutions and gels has been measured in the T range of −25 to 60 °C and is shown in Figure 2. Figure 2a shows the ion conductivity of NaG. The cycles show that there is a change in slope at about −10 °C, which is caused by the melting of the Na/diglyme complexes (see DSC in Figure 1c). Figure 2b shows the ion conductivity variation on cooling from 60 °C for NaG and the polymer electrolytes. The conductivity of NaG alone is slightly higher than any of the polymer mixtures, which is expected since the incorporation of polymer decreases the ion concentration and increases the viscosity. As a matter of fact, ion conductivities of the gels are quite high, taking into account their dimensional stability, and all polymer electrolytes would be adequate for further electrochemical testing. NaG and its gels show a change in slope in the σ­(T) curve at low T because of the crystallization of the liquid electrolyte.

**Figure 2:**
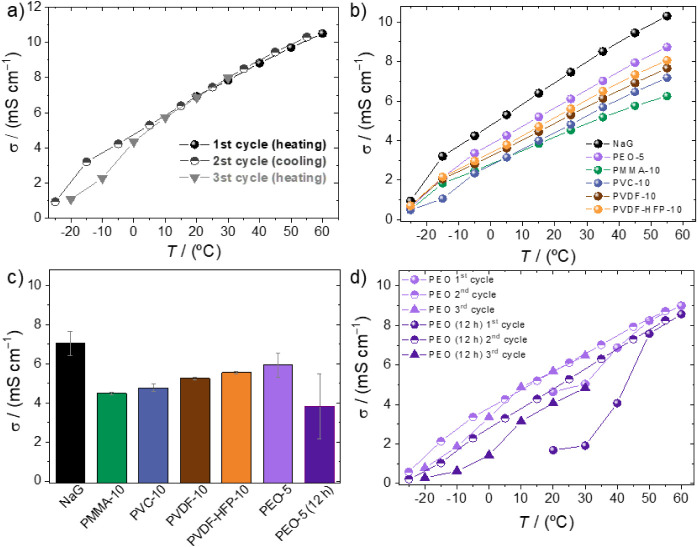
Ion conductivity of (a) NaG over the three temperature cycles from −25 to 60 °C, (b) the self-standing gels PVC-10, PVDF-10, PVDF-HFP-10, and PEO-5, and the solution of PMMA-10 in the cooling cycle (from 60 to −25 °C), (c) the polymer gels at 25 °C, and (d) PEO-5 and PEO-5 (12 h) over the three temperature cycles.

In Figure 2c, the ion conductivity at 25 °C is represented for all of the self-standing gel electrolytes (PVC-10, PVDF-10, PVDF-HFP-10, and PEO-5) and the dissolved PMMA-10. It is surprising that the latter, which is a liquid and not a gel, has lower conductivity than any of the other polymer gels with the same wt % (PVC-10, PVDF-10, and PVDF-HFP-10) all of which have similar ion conductivities of about 5 mS cm^–1^, which is remarkably high taking into consideration that they are self-standing. The lack of interaction of the PMMA carbonyl with Na^+^ characterized by FTIR, together with the low conductivity of this dissolution, suggests that the solubilization of PMMA in the electrolyte occurs by solubilization in the diglyme. The presence of PMMA then decreases the diglyme molecules available for the solubilization of the NaPF_6_ salt, producing liquid domains with larger local NaPF_6_ concentration and higher viscosity, which thus have lower ion conductivity.^3,31^ PEO gels show a decrease in ion conductivity during the first 12 h after preparation because of the crystallization of the complexes, and while the fresh PEO-5 gel is about 6.0 mS cm^–1^ (in comparison to 7.0 mS cm^–1^ NaG), once crystallized, it is about 3 mS cm^–1^. Figure 2d shows ion conductivity along three heating–cooling cycles of PEO-5 measured as prepared and after 12 h. The crystallization of the PEO complexes is seen in the low initial ion conductivity at 20 °C of PEO-5 (12 h), a conductivity that increases quickly as T rises and the crystals melt.

Figure 3a shows the self-diffusion coefficients at 25 °C of ^1^H and ^19^F, obtained by PFG solid-state NMR for PVC and PEO gels at different polymer concentrations, and the PMMA solution. The most notable feature of Figure 3a is that the diffusivity of both ^19^F and ^1^H depends mostly on the polymer wt %, and not so much on its chemical nature. D­(^19^F) decreases as the polymer concentration increases because of the local mobility restriction imparted by the polymer’s presence, and so does D­(^1^H). D­(^1^H) reflects not only the diglyme motion but, as the polymer ratio increases, also measures more and more the H in the polymer, and only the data with the lower polymer concentration are meaningful. The measurement of D­(^23^Na) can only be carried out at low polymer concentrations or higher T, because otherwise, relaxation times are too long. Anyway, as reported by other authors, D­(^23^Na) and D­(^19^F) are very close in solvated ionic liquids. This is checked in a set of PEO gels prepared with very low polymer concentration and measured at 30 °C, which appear in Figure 3b. In effect, D­(^23^Na) and D­(^19^F) are very similar and decrease slowly as the wt % of PEO increases, the transport number being close to 0.5 in all cases.

**Figure 3:**
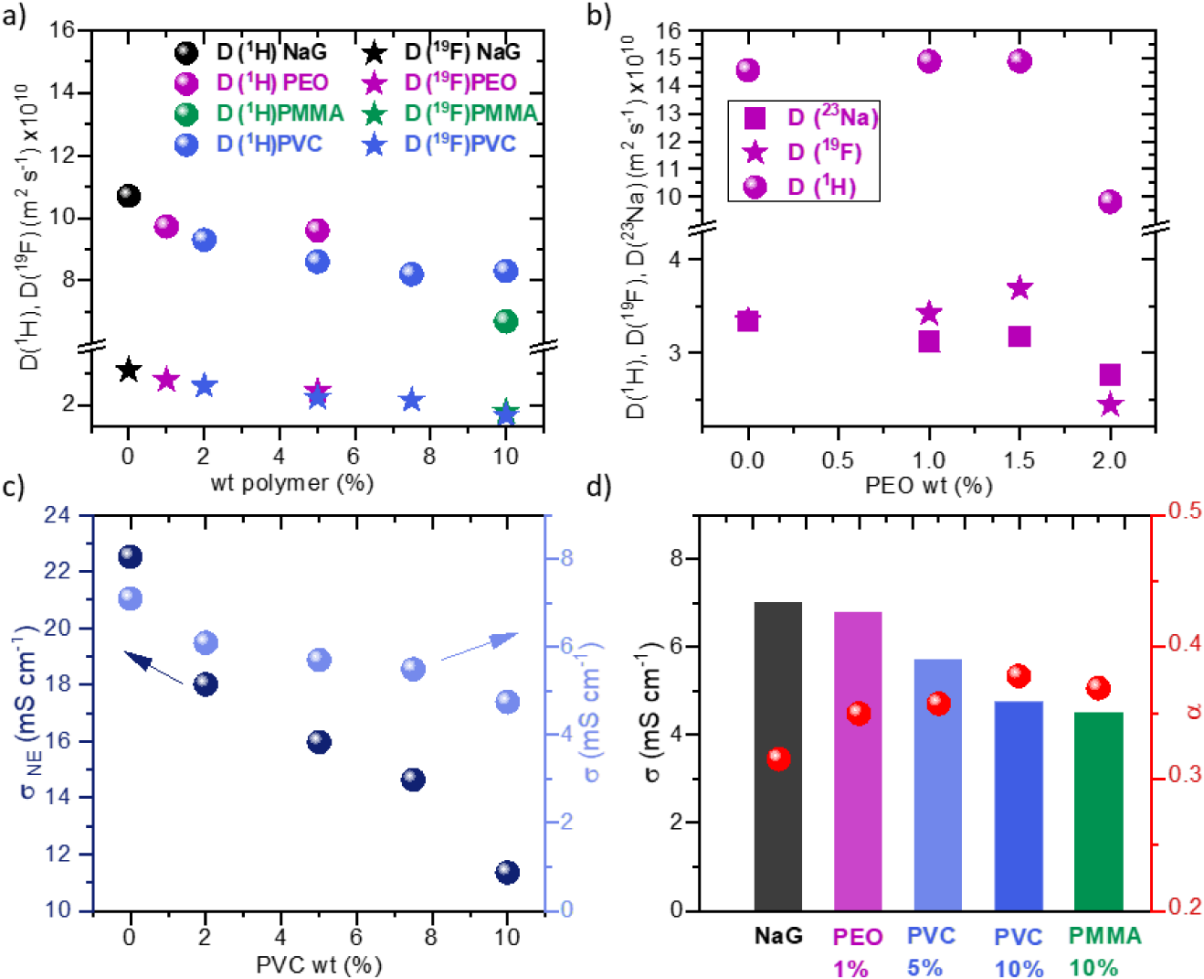
(a) Ion self-diffusion at 25 °C of ^1^H and ^19^F, showing the decrease in diffusivity with polymer wt %, (b) ion diffusivity at 30 °C, including ^23^Na, of PEO gels, (c) comparison between the σ and σ_NE_ at 25 °C of PVC gels, sand (d) σ and $\alpha =\frac{\sigma }{\sigma_{NE}}$ of several polymer electrolytes compared to NaG.

With the diffusion coefficients, it is possible to calculate conductivity values using the Nernst–Einstein equation (eq 1).

1 $$\sigma_{NE}=\frac{F^{2}}{RT}\cdot \underset{i}{\sum }n_{i}\alpha_{i}\cdot D_{i}$$

where *ni* and *Di* are the molar concentrations of the ion and its diffusion coefficient, respectively, and *F* is the Faraday constant. To do so, we have assumed that Na and F have the same diffusion coefficients, which is a good assumption in view of the literature and the results in Figure 3b. Figure 3c shows that, while the trends of σ and the σ_NE_ obtained from PFG NMR data are very similar, their actual values differ by about a 3-fold. The ratio *α* = *σ/σ*_*N*E_ is represented in Figure 3d for NaG and four different polymer mixtures: three gels, PEO-1, PVC-5, and PVC-10 (self-standing), and liquid PMMA-10. α varies only slightly from 0.31 for the pure NaG to 0.36 in the polymer electrolytes. It does not seem to depend on the nature of the polymer or even on its concentration; it is quite stable at values close to 0.35. Such low α values are not frequently seen in conventional electrolytes consisting of salt solutions or even in polymer gels, where *α* is usually close to 0.9, and it represents the dissociation degree of the salt. This behavior has been reported before in Na-glyme electrolytes, not only with PF_6_ salts but also with TFSI and FSI.^32^ As far as we know, this behavior remains unexplained.

### 3.3 Chemical Stability of the Polymer Gels

All gels were kept in the glovebox for periods of months to evaluate their mechanical and chemical stability. None of them showed deterioration in dimensional stability, but all the fluorinated polymer gels were seen to darken after several weeks in the glovebox (Figure 1a), denoting degradation of the electrolyte. This degradation was suspected to be dehydrofluorination, which has been reported before during the electrochemical cycling of Li^33^ and K cells^34^ when employing electrolytes containing PVDF and PVDF-HFP, and even in PVDF pipes in alkali media,^35^ by mere contact with NaOH (aq). This process is autocatalytic, as it produces HF, which further catalyzes the reaction. After several months, all PVDF gel electrolytes phase-separated into a fragile dark-brown gray film and transparent cubic crystals. Figure 4a shows pictures of degraded PVDF-5 and PVDF-15. In PVDF-5, a large cubic transparent crystal was formed, together with the brown-gray film. PVDF-15 forms a bilayer structure with a dark film on top and a continuous dark crystalline phase underneath. PVDF-10 is very similar to PVDF-15, except that instead of a continuous crystalline phase, a large number of tiny crystallites are observed. Since dehydrofluorination produces HF, it is most likely that the cubic crystals are NaF, which are formed in association with the presence of PVDF and are related to the initiation of the polymer chain degradation.

**Figure 4:**
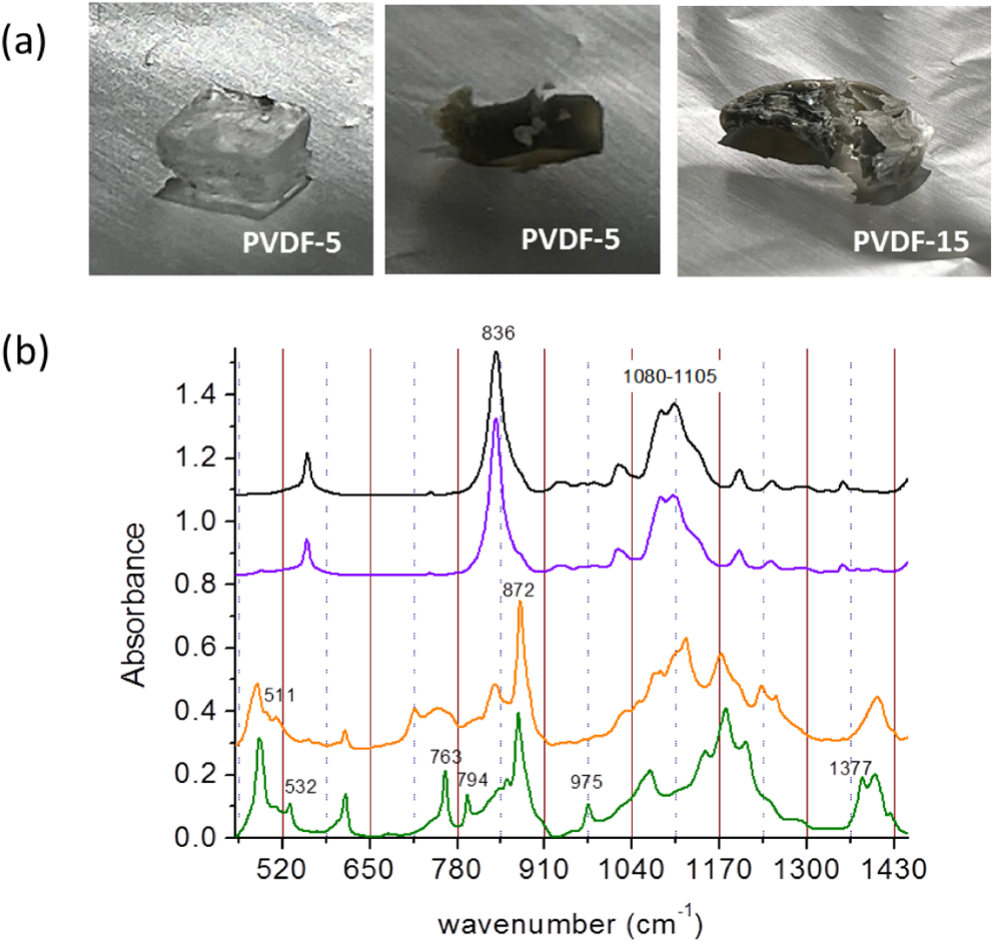
(a) Pictures of PVDF-5 and PVDF-15 degraded after several months in the glovebox, (b) FTIR in the region 500–1500 cm^–1^ of NaG (black), fresh (violet), and degraded (orange) PVDF-10 electrolyte and PVDF (green).

The FTIR spectra of PVDF-10, crystalline phase, and fragile dark film appear in Figure 4b, in comparison to PVDF, NaG, and fresh PVDF-10. PVDF-10 is almost identical to the liquid electrolyte NaG because of the low polymer concentration, and bands at 836 cm^–1^ and in the 1080–1105 cm^–1^ region dominate the spectra. The spectrum of degraded PVDF-10 (very similar to PVDF-5 and PVDF-15) shares many features with that of PVDF, indicating that the dark film is mainly PVDF, but some clear differences are also seen. First, in the degraded PVDF-10 film, there remain bands at 836 cm^–1^ and in the 1080–1105 cm^–1^ region because of the rest of the liquid electrolyte. Second, in comparison to PVDF, degraded PVDF-10 lacks the conformations found in the α crystalline phase, at 532, 763, 794, 974, and 1377 cm^–1^. At the same time, planar conformations, favored by double C=C bonding, and typical of the β phase appear: a band at 511 cm^–1^ and an increase in the relative intensity of the band at 872 cm^–1^. The increase of planar conformations in relation to coiled ones (such as those found in the α-phase) in dehydrofluorinated PVDF has been previously reported by other authors.^35,36^ The FTIR spectra in Figure 4b show, then, that PVDF gels in NaG are not chemically stable and are not adequate for this Na electrochemistry. Moreover, since dehydrofluorination occurs in alkaline conditions, before employing PVDF or PVDF-HFP binders, separators, or polymer gels in other electrochemistries, their true chemical stability should be checked.

Because of the degradation of the fluorinated gels, only the electrochemical behavior of PVC 10 wt % and PEO 5 wt % has been studied in the following section.

### 3.4 Electrochemistry in PW Cells with Na Metal and Hard Carbon as Anode

#### 3.4.1 Electrochemical Stability

In Figure 5 the electrochemical stability window of the gel electrolytes with PEO and PVC is shown by cyclic voltammetry. The gels were cycled in a range from 0 to 2.5 V and from 2.5 to 5 V vs Na^+^/Na. The PEO-based gel seems to passivate over the 3 cycles of CV and is judged to be stable within the 0 to 4 V vs Na^+^/Na range, similarly to the base liquid electrolyte (Figure 5a and 5b). In contrast, the PVC-based gel electrolyte shows a strong onset of reductive decomposition below 0.75 V (Figure 5c) and exhibits oxidative decomposition above 4.5 V vs Na^+^/Na (Figure 5d) These observations are consistent with a discoloration of the sodium reference/counter (inset Figure 5e). It should be noted that this reaction between the PVC-based gel and Na leads to an uncertainty in the potential of the reference electrode and, as such, the exact onset potential of reductive decomposition. This, however, does not change the conclusion that the reductive decomposition of the PVC-based gel renders it ill-suited to the cells discussed in this paper.

**Figure 5:**
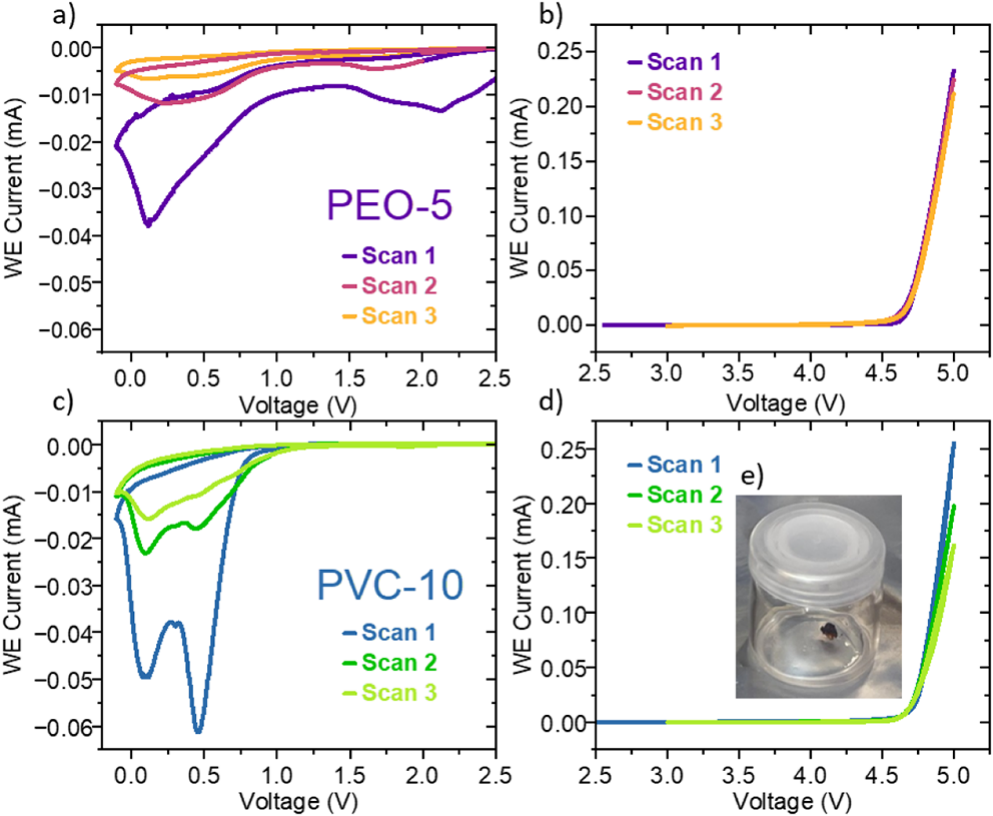
Electrochemical stability window on a Glassy Carbon working versus Na^+^/Na reference and counter electrodes: (a) PEO-5 reduction from 0 to 2.5 V, (b) PEO-5 oxidation from 2.5 to 5 V, (c) PVC-10 reduction from 0 to 2.5 V, (d) PVC-10 oxidation from 2.5 to 5 V, and (e) inset showing a PVC-10 picture after the measurement is finished.

#### 3.4.2 Cycling in PW//Na and PW//Hard Carbon Cells with NaG and PEO-5 Electrolytes

PEO-5 and NaG were used as electrolytes in PW||Na and PW||HC cells, and their performance was compared. Before doing so, it was checked that fresh PEO-5 (before crystallization of the Na/PEO/glyme crystalline complexes) and crystallized PEO-5 showed comparable results. It was found that, within the experimental error of these measurements, both were comparable.

Figure 6 shows the cycling of a PW||Na cell with the NaG electrolyte using a Celgard separator and the PEO-5 gel without a separator after 12 h of rest. The discharge capacity of the formation cycle (C/10) is similar between the NaG and PEO-5 (Figure 6a; error bars represent the range for 2 cells). The discharge capacity of the PEO-5 is observed to be lower over the proceeding 50 cycles at C/3, which is thought to be due to the vastly larger distance between the electrodes (1.1 mm for PEO-5 vs ∼20 μm for the NaG). Though a comparative study with similar electrode separation distances would be of great interest, doing so requires substantial engineering optimization, which is too extensive for the scope of this work. Despite the lower capacity at C/3 for the PEO-5 over the initial cycles, the capacity retention is seen to be similar between the gel and liquid electrolyte, and the Coulombic efficiency is observed to be slightly higher for the PEO-5 than the NaG. After 50 cycles at C/3, the NaG had an average discharge capacity of 132 mAh g^–1^ (79% capacity retention), and the PEO-5 had an average discharge capacity of 130 mAh g^–1^ (80% capacity retention).

**Figure 6:**
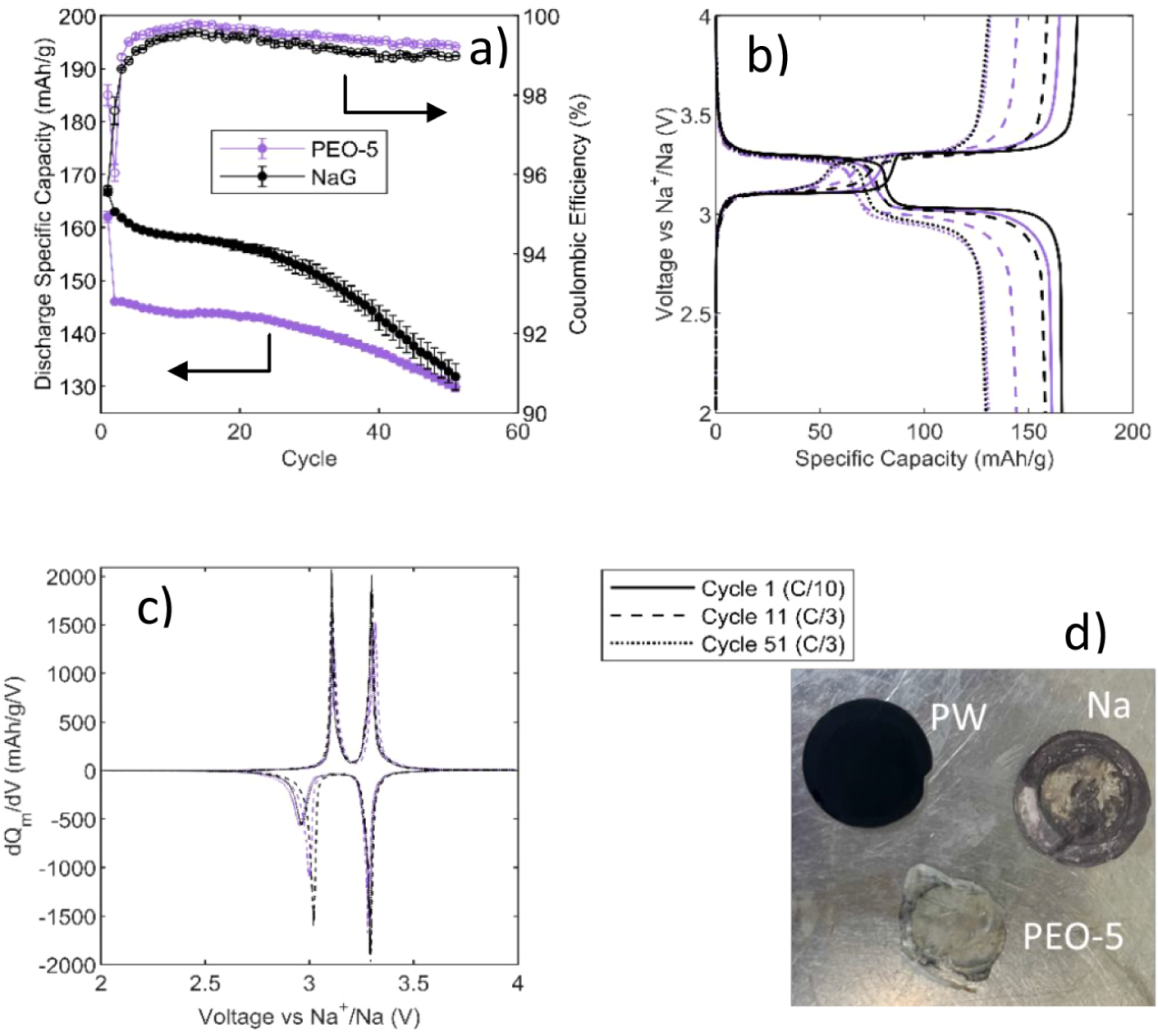
Electrochemical properties of NaG and PEO-5 in PW||Na cells over 51 cycles: (a) Discharge specific capacity and Coulombic efficiency vs cycle of NaG and PEO-5. Error bars represent the range for 2 cells, (b) specific capacity vs voltage plots for NaG and PEO-5 for the formation cycle (C/10) and cycles 11 and 51 (C/3), (c) dQ/dV plots (normalized to mass) for NaG and PEO-5 for cycles 11 and 51 (C/3), and (d) images of the separated components of the PW||Na cells after finishing the cycling experiment. In (b) and (c) only the results from 1 representative cell are shown.

The capacity–voltage plots and dQ/dV plots for representative cells are shown in Figure 6b and Figure 6c, respectively. The NaG and PEO-5 behave similarly, with no new electrochemical features observed in the PEO-5 cells. Polarization is observed to be slightly higher for the PEO-5, particularly in the discharge behavior on the lower plateau of the PW (i.e., reduction of the high-spin Fe). Though some of this polarization will be caused by the increased distance between electrodes in the PEO-5, the distinct increase in polarization in the lower plateau suggests that there may be increases in the interfacial resistance (e.g., poorer contact between the electrolyte and active materials or increased charge transfer resistance) as the PW transitions from a cubic to rhombohedral phase, which incurs a modest volume change. Nonetheless, it is remarkable that an ostensibly solid gel electrolyte performs similarly to a liquid electrolyte.

An interesting feature was discovered upon opening the cells after 51 cycles. After the experiments were finished, it was possible to mechanically separate the gel electrolyte from both the PW cathode and the Na anode, as shown in Figure 6d. This is because thanks to this crystallization, the gel becomes harder at the interface with the electrodes, allowing for a simple physical separation, with little or no gel sticking to the electrodes. During cell operation, the gel wets the electrode as a liquid, allowing for electrochemical performance similar to the pure NaG electrolyte, but upon crystallization, the electrolyte/electrode interface switches from stick to nonstick, which offers the possibility to isolate the three components of the cell.

Inspection of the gel electrolyte after separation from the electrodes showed that no coloring had occurred, and it retained its excellent mechanical properties, being dimensionally stable, tough, and flexible, and no visual evidence of dendrites or chemical degradation was seen. Note that no separator was needed in these cells, since PEO-5 was able to avoid contact during cell preparation and throughout the cycling experiment.

Figure 7 shows the galvanostatic cycling performance of PW||HC cells using the PEO-5 gel electrolyte in comparison with the liquid NaG electrolyte. As with the PW||Na cells, the formation capacity (C/10) is similar between the gel and liquid electrolytes, but a lower capacity is observed at C/3 (Figure 7a). The Coulombic efficiency between the electrolytes was similar to an initial Coulombic efficiency of around 80% (81.7% for NaG and 77.4% for PEO-5). By the third cycle, the Coulombic efficiency was above 99% for both electrolytes. After 50 cycles at C/3, PEO-5 showed an average discharge capacity of 107 mAh g^–1^ (83% capacity retention) compared to 118 mAh g^–1^ for NaG (87% capacity retention).

**Figure 7:**
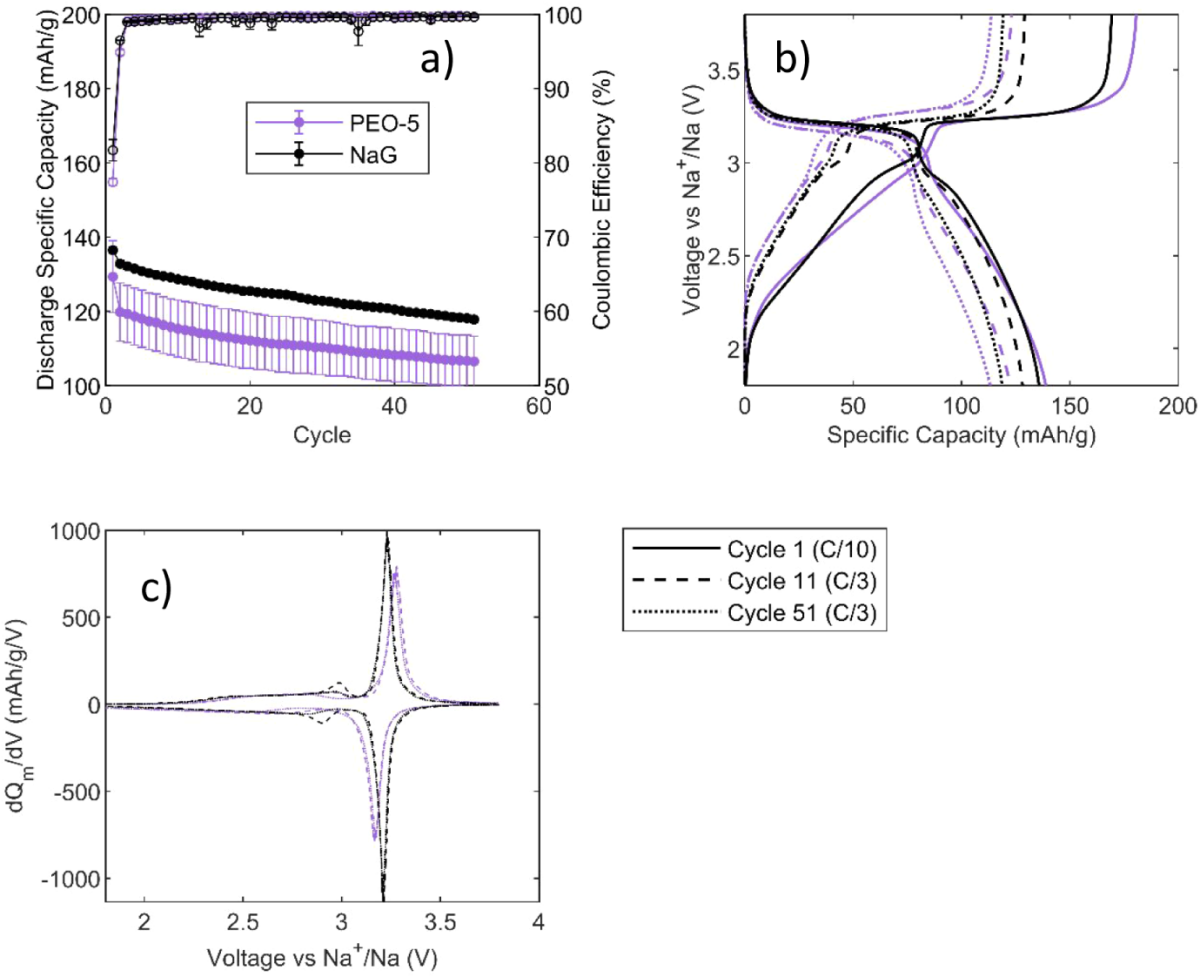
Electrochemical properties of NaG and PEO-5 in PW||HC cells over 51 cycles: (a) Discharge specific capacity and Coulombic efficiency vs cycle of PEO-5 and NaG. Error bars represent the range from 2 cells, (b) specific capacity vs voltage plots for PEO-5 and NaG for the formation cycle (C/10) and cycles 11 and 51 (C/3), and (c) dQ/dV plots (normalized to PW electrode mass) for cycles 11 and 51 for NaG and PEO-5. For (b) and (c) only the cycling for 1 representative cell is shown.

While the capacity-voltage plots (Figure 7b) are similar between the NaG and PEO-5, there are some subtle differences in the dQ/dV plots (Figure 7c). The main difference is the polarization, which is most evident in the separation of the peak at 3.3 V (corresponding to the low-spin Fe redox and plateau region of the HC). Polarization is observed to be higher for the PEO-5 than for the NaG, which again may be due to the larger separation between electrodes but may also be the result of increased interfacial resistance. A small peak is evident for the NaG, centered around 3 V, which is almost certainly an artifact of cell balancing; here, the anode capacity is slightly lower, and so the HC reaches the plateau region while the PW is still on the lower plateau. In general, however, aside from increased polarization, the electrochemical performance of the PEO-5 and NaG is remarkably similar. Considering the promising capacity retention and first-cycle Coulombic efficiency, and keeping in mind that the cell setup is, for the most part, unoptimized, these results show that the PEO-5 gel electrolyte is applicable as a separator-free, solid-like electrolyte material for SIBs.

## 4 Conclusions

The most common polymers used to turn into solid-liquid electrolytes are tested with the SIB electrolyte NaPF_6_ in diglyme (1.1 m). PMMA failed to achieve a gel electrolyte in this system, at least at contents under 10 wt % (note that increasing polymer content would lead to a notable decrease in conductivity and, hence, in electrochemical performance). Fluorinated polymers such as PVDF and PVDF-HFP provide self-standing gel electrolytes from 10 wt % polymer content. However, these gels become brownish after a few weeks because of the dehydrofluorination of the polymers in the Na electrolyte. Self-standing gels from PVC are obtained from 7 wt % content onward and remain stable over a long period of time. Unfortunately, the electrochemical stability of these PVC gels is not enough to be useful for this application. On the contrary, 5 wt % UHMW PEO gels, which are solid, stable, and homogeneous, present a wide electrochemical window and show similar performance in PW||Na half-cells and PW||HC full cells as the baseline electrolyte. In addition, the PEO-5 hard gel has several advantages: it avoids leaks of the flammable electrolyte in case of an accident, makes the use of a separator unnecessary, and offers new recycling paths for electrochemical cells, which permit the separation of components and their individual recycling.

## Supplementary Material



## Notes

The SI file contains: a picture of the electrochemical cell; FTIR spectra of different gels; DSC melting endotherms of PEO, NaG, and gels; and long-term stability image of the PEO gel (PDF)
