# Supplementary material for: Na Battery Electrolytes Prepared by Dissolution of Commercial Polymers in NaPF6–Diglyme
Source: ACS Appl Polym Mater. 2026 Apr 16;8(9):6843–53. doi: 10.1021/acsapm.6c00803 (PMC13162201; doi:10.1021/acsapm.6c00803)
Supplement: Supplementary file 1 [file ap6c00803_si_001.pdf]

Supporting Information

Na battery electrolytes prepared by dissolution of commercial polymers in $NaPF_6$ - diglyme

Ángela Campo[1], Nuria García[1], Ana López-Cudero[1], Aram Hall[2], Reza Younesi[2] and Pilar Tiemblo[1]*

[1]Instituto de Ciencia y Tecnología de Polímeros, ICTP-CSIC, Calle Juan de la Cierva 3, 28006 Madrid, Spain

[2]Department of Chemistry – Ångström Laboratory, Uppsala University, Uppsala, 75121 Sweden

angcampo@ictp.csic.es, ngarcia@ictp.csic.es, ana.cudero@ictp.csic.es, charles-aram.hall@kemi.uu.se, reza.younesi@kemi.uu.se, ptiemblo@ictp.csic.es

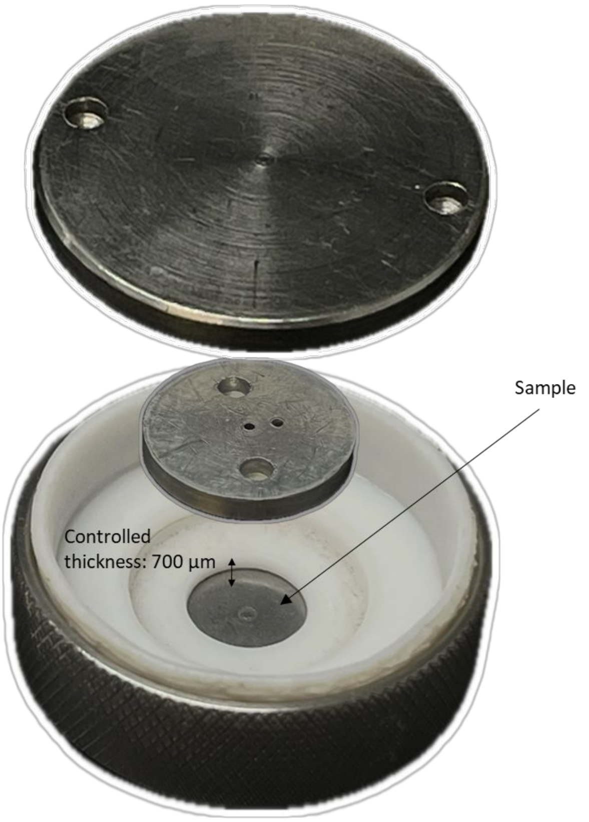

Figure S1: Picture of the cell employed to measure the liquid, gel and solid electrolytes.

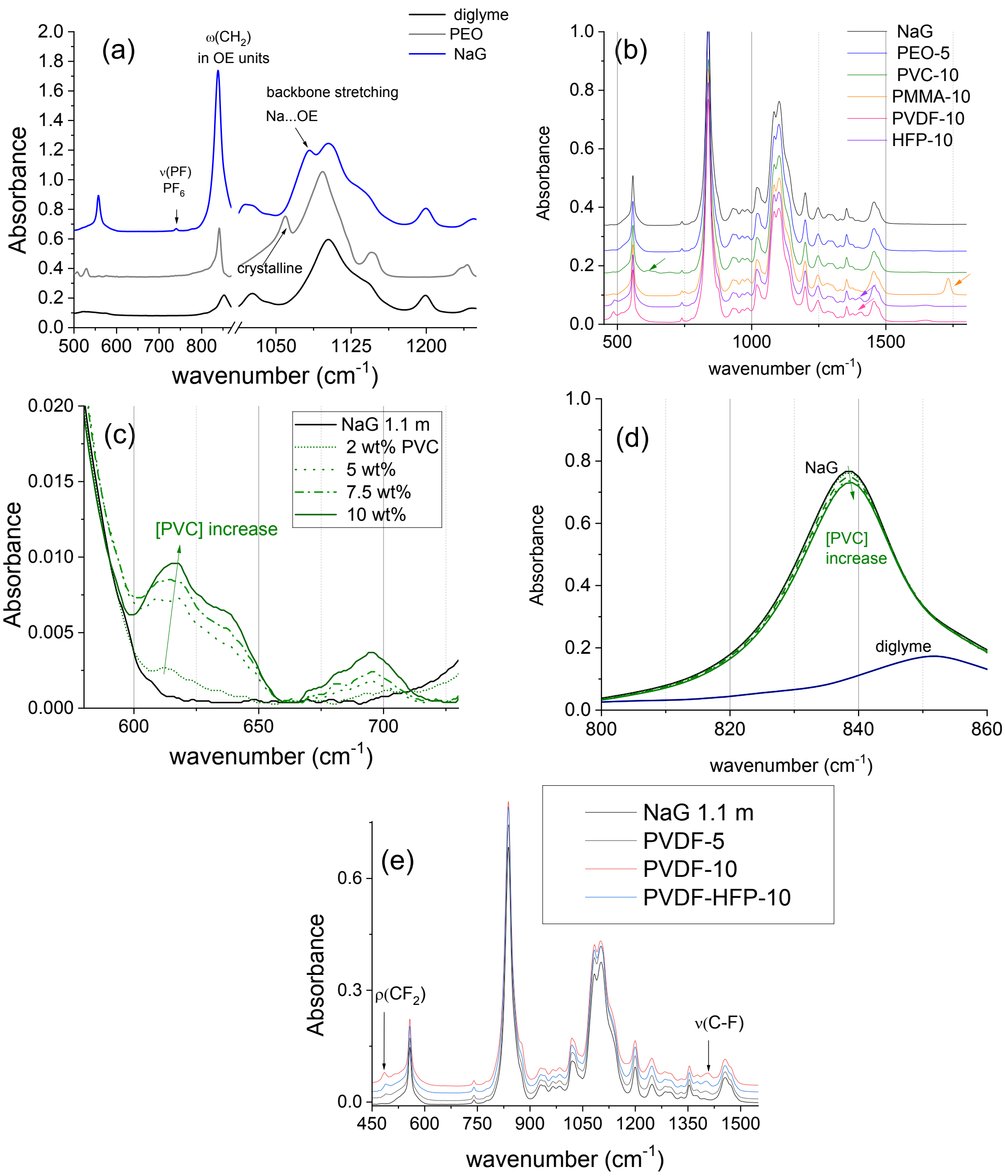

Figura S2. FTIR spectra of (a) diglyme, PEO and NaG, (b) NaG (black), PVC-10 (green), PEO-5 (blue), PMMA-10 (orange), PVDF-10 (pink) and PVDF-HFP-10 (violet), with arrows pointing at bands characteristic of the polymers, (c) PVC gels within the region associated to the ν(C-Cl) , (d) diglyme, NaG and the PVC gels within the region associated to ω(CH_2), and (e) NaG, PVDF-5, PVDF-10, and PVDF-HFP-10.

Figure S2a collects the FTIR spectra of diglyme, NaG and PEO, highlighting relevant vibrations. **Figure S2b** shows the spectra of the self-standing polymers gels and the PMMA dissolution. Clear bands of the dissolved polymers are seen: the ν (C=O) of PMMA at 1732 cm^{-1}, the ν(C-Cl) of PVC in the range 600-700 cm^{-1}, PVDF and PVDF-HFP ν(C-F) at 1400 cm^{-1}.

Figure S2d shows how the ν(C-Cl) of PVC is more intense as the concentration of the polymer in the gel increases. No modifications in that band are perceptible in the FTIR. **Figure S2c** shows the ω(CH_2) of pure diglyme, diglyme in NaG and the PVC gels. All the polymers used in this work have IR bands at about 840 cm^{-1}, very intense in PVDF and PVDF-HFP, and of low intensity in PMMA and PVC. PVDF, PVDF-HFP and PEO downshift the ω(CH_2) band to 838 cm^{-1}

(not shown), what can merely be an additive contribution of the pure polymer bands in this region, which are strong in these three polymers. However, PVC produces an upshifting, slight but clear, to 839 cm^{-1}, as the concentration of polymer increases, accompanied by a decrease in intensity and width of the band (becoming closer to the pure diglyme band). This anomalous modification of the band, opposite to additive contribution of pure PVC bands, may suggest that PVC interacts with the Na cation to a certain extent, competing with diglyme and decreasing its implication in the complexation (more “free” diglyme). **Figure S2e** shows the FTIR spectra of PVDF-5, PVDF-10 and PVDF-HFP-10. In all of them bands characteristic of the CF_2 group are seen.

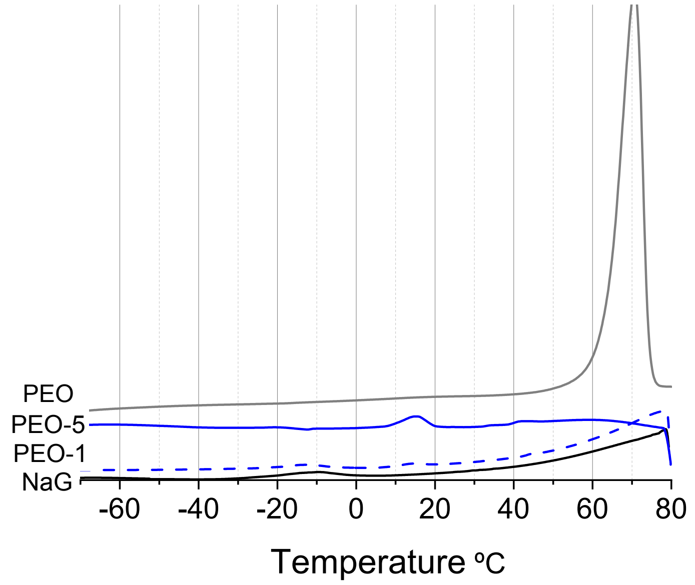

Figure S3. DSC heating runs of PEO, PEO-5, PEO-1, and NaG.

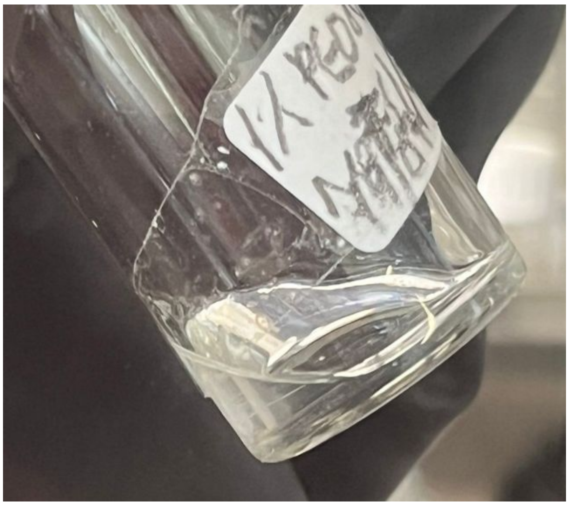

Figure S4. Image of PEO gel in TFSI 1 m in diglyme with 1 wt% of polymer after several weeks.
